# Sarcosine dehydrogenase as an immune infiltration-associated biomarker for the prognosis of hepatocellular carcinoma

**DOI:** 10.7150/jca.89616

**Published:** 2024-01-01

**Authors:** Haixiang Xie, Kejian Yang, Chongjiu Qin, Xin Zhou, Junqi Liu, Jusen Nong, Jianzhu Luo, Yongguang Wei, Huasheng Hua, Chuangye Han, Xiwen Liao, Chengkun Yang, Hao Su, Guangzhi Zhu, Xinping Ye, Tao Peng

**Affiliations:** 1 Department of Hepatobiliary Surgery, The First Affiliated Hospital of Guangxi Medical University, Nanning, 530021, Guangxi Zhuang Autonomous Region, People's Republic of China.; 2 Guangxi Key Laboratory of Enhanced Recovery after Surgery for Gastrointestinal Cancer, 530021, Nanning, People's Republic of China.; 3 Key Laboratory of early Prevention & Treatment for regional High Frequency Tumor (Guangxi Medical University), Ministry of Education, Nanning, 530021, Guangxi Zhuang Autonomous Region, People's Republic of China.

**Keywords:** Hepatocellular carcinoma, Sarcosine dehydrogenase, Biomarkers, Immune infiltration, Prognostic

## Abstract

This study was aimed to investigate the prognostic value and clinical significance of sarcosine dehydrogenase (SARDH) in hepatocellular carcinoma (HCC) and to explore the underlying mechanisms. The Cancer Genome Atlas (TCGA), Gene Expression Omnibus (GEO), HPA and CPTAC databases were adopted to analyze the expression of SARDH mRNA and protein between normal liver tissue and HCC, and examine their relationship with clinicopathological features. Kaplan-Meier analysis, Cox regression, as well as nomogram were adopted to explore the prognostic value of SARDH in HCC. Gene Ontology (GO), Kyoto Gene and Genome Encyclopedia (KEGG) together with Gene Set Enrichment Analysis (GSEA) were adopted to analyze the molecular mechanisms and biological functions of SARDH in HCC; while MethSurv, STRING, GeneMANIA, TIMER database data and single-sample gene set enrichment analysis (ssGSEA) algorithm were used for other bioinformatic analysis. Furthermore, immunohistochemistry was used to verify the expression of SARDH. Compared to normal liver tissue, SARDH expression was markedly lower in HCC. A lower SARDH expression was linked with Pathologic T stage (T3&T4), pathologic stage (Stage III&IV), and histologic grade (G3&4), which further indicates worse prognosis. Besides, results of bioinformatic analysis proved that SARDH expression was correlated with immune infiltration. In addition, SARDH hypermethylation was related to a poorer prognosis. SARDH expression was related to several key genes in the Ferroptosis pathway.

## Introduction

In the world, primary liver cancer has ranked the 6^th^ of the most commonly diagnosed cancers as well as the 3^rd^ most deadly malignancy. Recent data has shown an increase in the incidence and mortality rate of liver cancer compared to previous published studies 1, 2. The economic burden of liver cancer is substantial, accounting for 6.5% of the global economic cost of cancer, which is the fourth highest of all cancers 3. Hepatocellular carcinoma (HCC) constitutes 85-90% of primary liver cancers 4. Non-alcoholic steatohepatitis, infection of hepatitis virus as well as alcohol-related hepatitis, are associated with development of HCC 5. Surgical resection has been recognized as the primary treatment for HCC 6. However, its high recurrence rate poses a major problem 7. The emergence of immune checkpoint inhibitors has revolutionized immunotherapy for HCC 8, with recent clinical studies showing promising prognosis with combined immunotherapy 9-11. However, the response rates of different checkpoint inhibitors in immunotherapy have remained unsatisfactory 12, 13. It is well-established that the tumor microenvironment (TME) exerts a crucial effect on immunotherapy in liver cancer 4. Therefore, it is particularly important to understand the potential mechanism, TME and new targets of immunotherapy in HCC.

SARDH is located on chromosome 9q34.2 and is an important enzyme in mammals, playing a vital role in catalyzing the oxidative demethylation of sarcosine to glycine metabolism 14, 15. It can function in conjunction with folic acid in the liver 16. SARDH is a suppressor gene for incidental colorectal cancer, high expression of which suppresses the biological functions of colorectal cancer cell lines 17. Notably, SARDH is strongly associated with DNA methylation, serving as a prognostic biomarker for renal cell carcinoma, significantly correlating with clinicopathological features 18. In prostate cancer, SARDH plays a significant prognostic role by altering the aggressiveness of cancer cells through interaction with TMEFF2 19. Prior studies have found significant downregulation of SARDH expression in HCC tissues, but the molecular mechanisms of SARDH pathogenesis in HCC and its correlation with prognosis have not been fully analyzed 20.

Therefore, we conducted bioinformatics analysis across various HCC databases to explore the correlation of SARDH expression in HCC with its prognosis value, potential molecular mechanisms, as well as immune cell infiltration.

## Materials and Methods

### Data Collection

Data on SARDH expression in the specimens of 374 HCC tissues and 50 paraneoplastic tissues and clinical features were collected from The Cancer Genome Atlas (TCGA) (https://portal.gdc.cancer.gov). To increase the specimens of paraneoplastic tissues, we extracted SARDH expression data from the UCSC XENA (https://xenabrowser.net/datapages/) database, among which the pan-cancer / normal tissue data were acquired from TCGA and the Genotype-Tissue Expression (GTEx) datasets, respectively. Meanwhile, we downloaded Gene Expression Omnibus (GEO) datasets (GSE14520, GSE62232, GSE136247, GSE121248) from the National Center for Biotechnology Information (https://www.ncbi.nlm.nih.gov/). HCC information from TCGA is shown in **Table 1.** GSE14520 related information is shown in **Table 2**.

### SARDH protein expression

We used the Human Protein Atlas (HPA, https://www.proteinatlas.org), an online site that provides immunohistochemical images for protein expression analysis 21 and The Clinical Proteomic Tumor Analysis Consortium (**CPTAC**) database of UALCAN (https://ualcan.path.uab.edu/analysis-prot.html) 22, to examine the SARDH protein expression in HCC compared to normal hepatocytes.

### Prognostic analysis of SARDH in HCC

Patients with HCC in the TCGA and GSE14520 datasets were adopted in this study and classified into low / high SARDH expression group according to the median of SARDH expression. The Kaplan-Meier curve was applied to explore the prognostic significance of SARDH expression. Univariate & multivariate Cox regression analyses were employed to test the predictive value of different clinicopathological characteristics. The significant prognostic variables (p < 0.1) in the univariate Cox regression were included in the multivariate Cox regression analysis.

Prognostic analyses (overall survival [OS], relapse-free survival [RFS], progression-free survival [PFS], as well as disease-specific survival [DSS]) of SARDH were performed for HCC using the online site of Kaplan-Meier (K-M) Plotter (https://kmplot.com/analysis/) 23. Additionally, the GEPIA (http://gepia.cancer-pku.cn/) database was employed for the prognostic analysis (OS) of SARDH in HCC 24.

To investigate the prognostic significance of SARDH and its clinicopathological features, SARDH expression, T-stage, M-stage, and N-stage were included in nomogram. The R package "rms" was utilized to construct the nomogram to predict OS. The predictive ability of nomogram was assessed using calibration curves.

A risk scoring system was constructed based on SARDH expression. Risk score = 9 - SARDH expression. Patients in TCGA were classified into high/ low-risk groups using the median of risk score. Besides, the K-M curves were generated using the survminer package. Time-dependent receiver operating characteristic (**ROC**) curves were analyzed using the “timeROC” package. The final visualization of the resulting data was performed with the “ggplot2” package.

### Differential expression analysis

The differentially expressed genes (DEGs) with adjusted P < 0.05 and |log2Foldchange |>1.5 in TCGA between the groups with high and low SARDH expression were analyzed using the "DESeq2" package. Correlations between the expression of the top 5 most up- / down-regulated DEGs and SARDH expression were examined using Spearman correlation analysis. All DEGs were presented as volcano plots.

### Functional enrichment analysis

Gene Ontology (GO), Kyoto Gene and Genome Encyclopedia (KEGG) analysis as well as GSEA (c2.cp.all.v2022.1. Hs. symbols. gmt) were performed on DEGs using the “clusterProfiler” package. The results were visualized using the ggplot2 package, and significance was determined using adjusted p-values <0.05 as well as false discovery rates (FDR) <0.25.

### DNA methylation

To analyze the relationship between SARDH and DNA methylation in HCC, MethSurv (https://biit.cs.ut.ee/methsurv/), an effective tool for survival analysis on DNA methylation patterns, was carried out 25.

### Protein interactions and gene interactions

Protein interactions were analyzed using STRING (https://cn.string-db.org) 26 with a minimum interaction score of 0.7, and the protein-protein interactions (PPI) network of SARDH proteins and 15-related proteins were obtained. Meanwhile, gene interactions and their gene functions were analyzed using GeneMANIA (http://genemania.org) 27.

### Immune infiltration

Using the GSVA package, the relative enrichment scores of 24 immune cells in HCC were calculated according to the ssGSEA algorithm. The association of SARDH expression with the infiltration level of 24 immune cells was assessed using Spearman analysis 28, 29. Furthermore, immune infiltration of groups with a high / low SARDH expression were compared using the Wilcoxon method. Meanwhile, the relationship of SARDH expression with the infiltrating level of 6 immune cells (CD8+ T cells, neutrophils, CD4+ T cells, dendritic cells, B cells and macrophages) was examined with the help of TIMER (https://cistrome.shinyapps.io/timer/). Additionally, "ESTIMATE" package was adopted to calculate the immunoscore, stromal score, as well as estimated score for HCC samples in TCGA; the correlation between the three scores and SARDH expression was analyzed using the SangerBox (http://vip.sangerbox.com/) platform.

### Immunohistochemistry (IHC)

To verify SARDH expression, 32 pathological specimens were collected from patients diagnosed with HCC in the First Affiliated Hospital of Guangxi Medical University. Inclusion criteria: 1. Patients with primary hepatocellular carcinoma treated for the first time; 2. Patients treated with partial hepatectomy; 3. Patients who have not undergone interventional therapy, targeted therapy and immunotherapy before surgery. Exclusion criteria: Patients with a history of other tumors besides hepatocellular carcinoma. This study had acquired the approval of the Ethics Committee of the first affiliated hospital of Guangxi Medical University before specimen collection, was conducted in line with the Declaration of Helsinki. Approval Number: NO.2023-E534-01. Written informed consent was provided by each patient.

Paraffin sections of tumor tissue and corresponding paracancerous tissue were fixed in 10% formalin, made into 4-μm sections and then fixed on slides. Following xylene dewaxing, we performed hydration with gradient concentrations of ethanol (100%, 95%, 85%, 75%), antigen repair (95°C), blockade of endogenous peroxidase activity with H_2_O_2_ (0.3%, room temperature), and then incubation with SARDH primary antibody (Proteintech, 22762-1-AP, 1:200) at 4°C overnight. Next, samples were incubated with secondary antibody for 30 minutes, followed by staining with diaminobenzidine (DAB) solution, re-staining with hematoxylin, dehydration with a gradient concentration of ethanol (75%, 85%, 95%, 100%), and blocking. IHC was performed by two pathologists who were blind to this study. The staining intensity was rated as follows: (1) 0: no color; (2) 1: light yellow; (3) 2: yellow; (4) 3: brown; while the staining degree was determined by the percentage of positive cells: (1) 0: ≤5%; (2)1: 5-25%; (3) 2:26-50%; (3) 3:51-100%. Final staining score was calculated on a scale of 0 to 6 17, among which 0-2 and 3-5 were defined as a low and a high expression, respectively.

### Statistical analysis

Data analysis was performed using R (version 4.2.1). Wilcoxon rank sum test was adopted to assess the relationship of SARDH expression with HCC / normal liver tissues and clinicopathological features. Student's t-test was used to assess SARDH expression in unpaired and paired tissues. Chi-Square test and Yates's correction were adopted to compare the categorical variables between groups. A P-value < 0.05 was indicative of statistical significance.

## Results

### Expression of SARDH is down-regulated in HCC at the mRNA level

**Figure 1:**
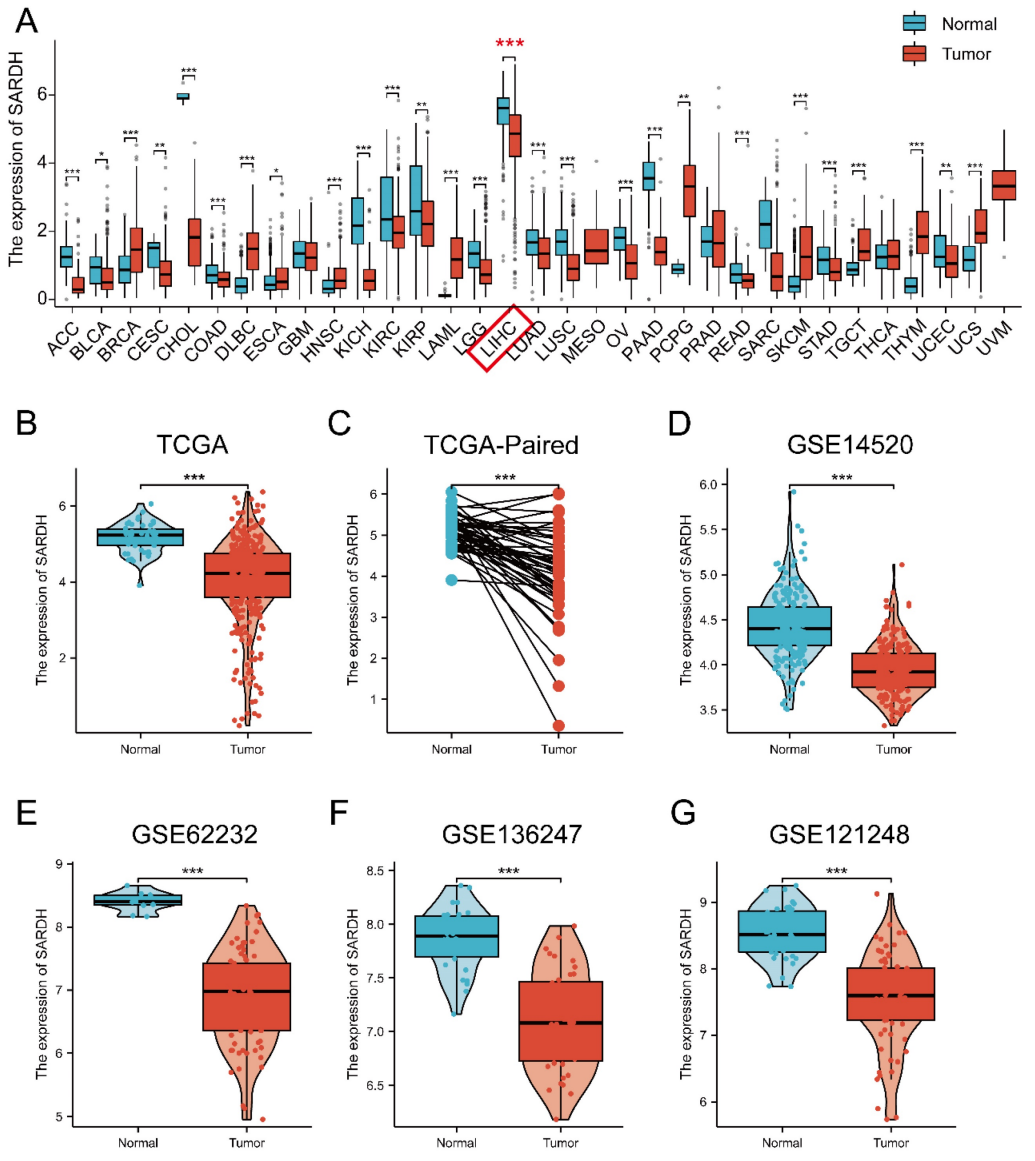
Pan-cancer analysis of SARDH and expression of SARDH in HCC and adjacent normal tissues. (A) Pan-cancer analysis of SARDH in TCGA and GTEx databases. (B) TCGA database of HCC and unpaired normal liver tissues. (C) TCGA database of HCC and paired normal liver tissues. (D) GSE14520. (E) GSE62232. (F) GSE136247. (G) GSE121248. ns: p ≥ 0.05; *p < 0.05; **p < 0.01; ***p < 0.001.

Pan-cancer analysis from TCGA and GTEx databases revealed remarkable differences in SARDH expression across 27 tumor tissues (**Figure 1A**). Particularly, SARDH mRNA expression was significantly downregulated in HCC, as demonstrated by the analysis of the TCGA-LIHC cohort and HCC paired samples (**Figure 1B, C**). These findings were further validated in HCC databases of GSE14520, GSE62232, GSE136247, and GSE121248 (**Figure 1D-G**).

**Figure 2:**
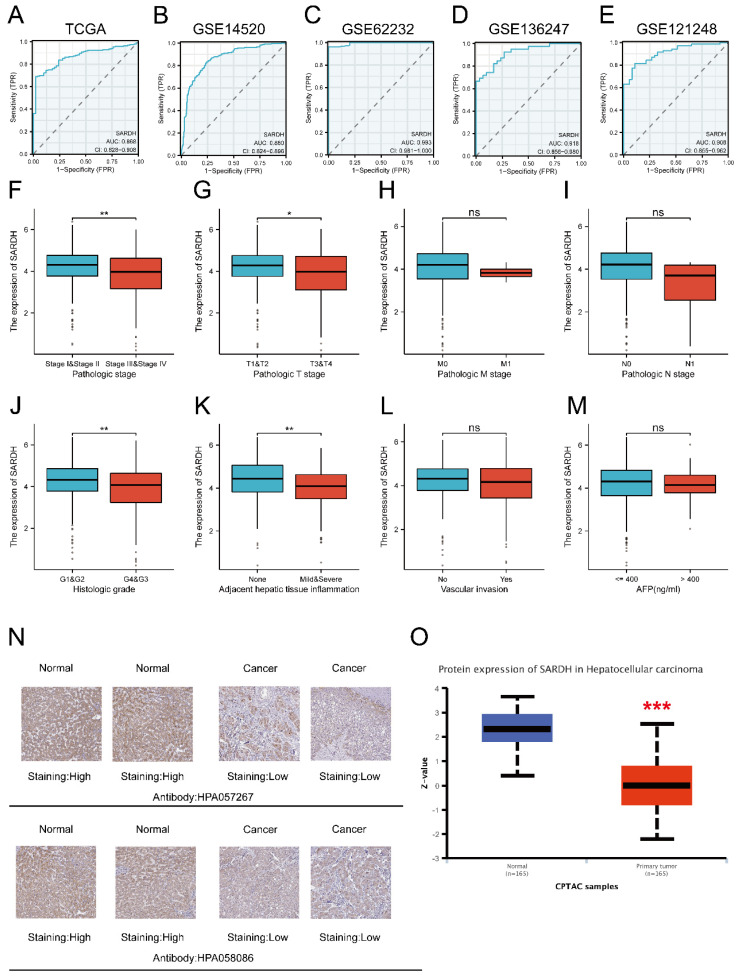
ROC analysis and relationship between SARDH and clinicopathological features. (A) ROC of TCGA. (B) ROC of GSE14520. (C) ROC of GSE62232. (D) ROC of GSE136247. (E) ROC of GSE121248. (F) Pathological stage. (G) T stage. (H) M stage. (I) N stage. (J) Histologic grade. (K) Adjacent liver tissue inflammation. (L) Vascular invasion. (M) AFP. Typical immunohistochemical images of SARDH expression in HCC tissues and normal liver tissues from the HPA database (N) and SARDH protein expression in HCC tissues and normal liver tissues from the CPTAC database in the UALCAN website (O). AFP, alpha-fetoprotein. ns: p ≥ 0.05; *p < 0.05; **p < 0.01; ***p < 0.001.

ROC curves were employed to evaluate the diagnostic efficacy of SARDH, with the area under curve (AUC) > 0.8 and p < 0.05 indicative of a better diagnostic performance 30, 31. Results of ROC analysis were presented as follows: TCGA (AUC=0.868); GSE14520 (AUC=0.860); GSE62232 (AUC=0.993); GSE136247(AUC=0.918); GSE121248 (AUC=0.908) (**Figure 2A-E**).

### Association of SARDH expression with clinicopathological features

A significant association was demonstrated between SARDH and clinicopathological features, including pathologic T stage (T3&T4) (P=0.002), pathologic stage (Stage III & IV) (P=0.002), adjacent hepatic tissue inflammation (mild & severe) (P=0.008), and histologic grade (G3&4) (P=0.022) (**Table 1**, **Figure 2F-M**).

### Expression of SARDH is down-regulated in HCC at the protein level

SARDH protein expression was evaluated using typical IHC images of HCC and normal liver tissues downloaded from HPA website. Notably, SARDH protein expression levels were significantly decreased in HCC. In addition, SARDH proteomic expression profile obtained from the CPTAC database confirmed the downregulation of SARDH protein expression compared to normal liver tissue (**Figure 2N-O**).

### Low SARDH expression correlated with worse prognosis

**Figure 3:**
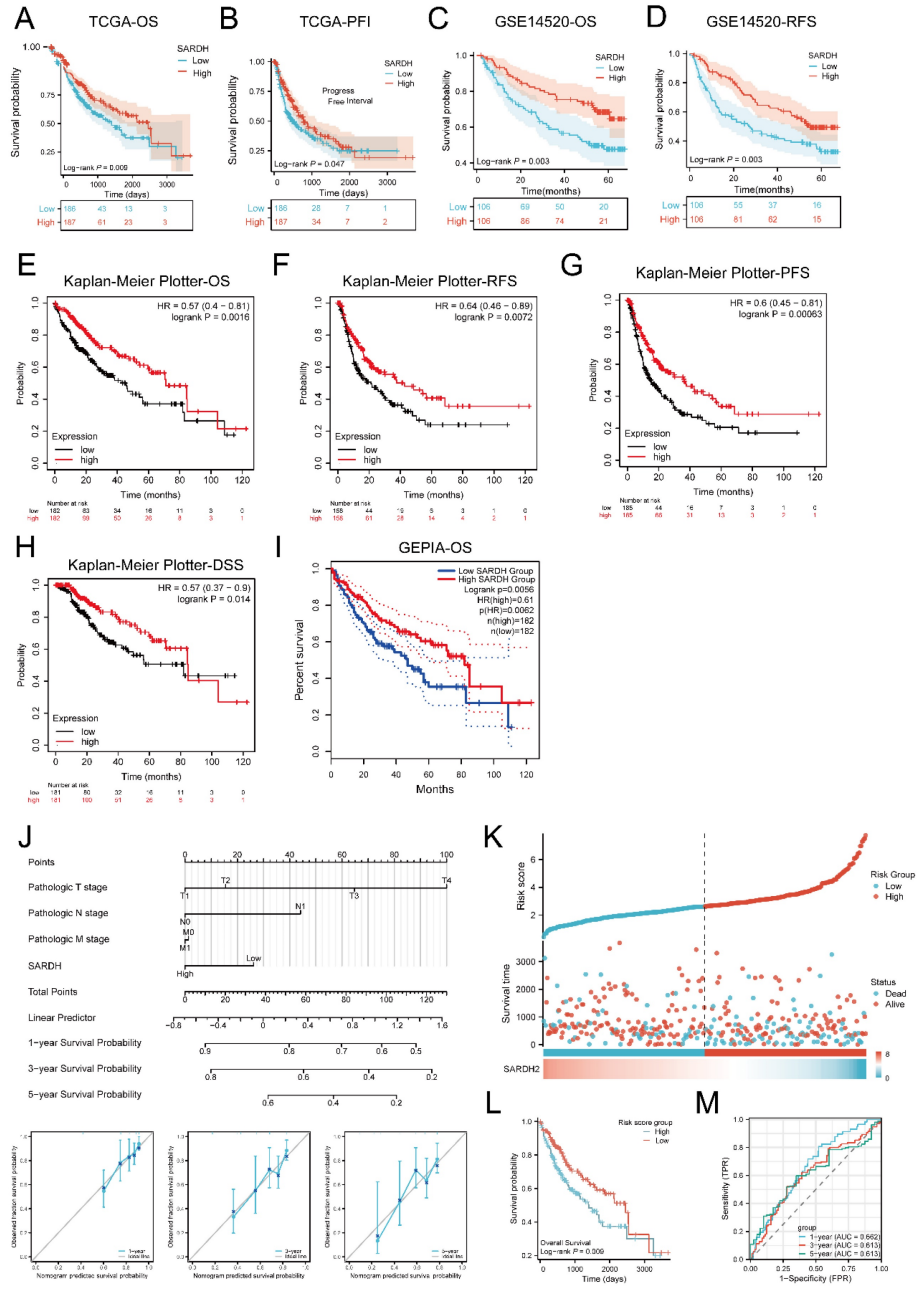
Kaplan-Meier analysis of SARDH in HCC. (A) Survival curves of OS in TCGA. (B) Survival curves of PFI in TCGA. (C) Survival curves of OS in GSE14520. (D) Survival curves of RFS in GSE14520. (E) Survival curves of OS in Kaplan-Meier Plotter. (F) Survival curves of RFS in Kaplan-Meier Plotter. (G) Survival curves of PFS in Kaplan-Meier Plotter. (H) Survival curves of DSS in Kaplan-Meier Plotter. (I) Survival curves for OS in GEPIA. (J)A nomogram and calibration curves for prediction of 1, 3, 5 year overall survival rates of patients with HCC. (K) Risk score, survival time distribution, and gene expression heat map of SARDH in TCGA. (L) Kaplan-Meier survival analysis of OS between high-risk and low-risk groups. (M) Time-dependent ROC curve for risk score models in TCGA. OS, Overall Survival. PFI: Progression Free Interval. RFS, Recurrence Free Survival. PFS, Progression Free Survival. DSS, Disease Free Survival.

Results demonstrated that in terms of OS(P=0.009) as well as PFI (P=0.047), low SARDH expression correlated with worse prognosis compared to the SARDH high expression group (**Figure 3A-B**). Similar results were obtained from Kaplan-Meier analysis in the GSE14520 database, where low SARDH expression was associated with inferior OS and RFS (P=0.003) (**Figure 3C-D**). In addition, analysis of OS, RFS, and PFS among patients with using K-M Plotter together with GEPIA2 revealed that low SARDH expression was strongly related to a poor prognosis (**Figure 3E-I**).

Further investigation via univariate and multivariable analyses in TCGA demonstrated that decreased SARDH expression (adjusted HR = 0.572, 95% CI = 0.363-0.902, p = 0.016), T3 stage (adjusted HR = 2.921, 95% CI = 1.748-4.882, p < 0.001), and T4 stage (adjusted HR = 6.125, 95% CI = 2.151-17.443, p < 0.001) were prognostic factors.

A nomogram incorporating SARDH expression, T-stage, M-stage, and N-stage was constructed to predict OS in HCC, with the contribution of each variable expressed by the length of the scale. The nomogram showed a good predictive performance, as demonstrated by calibration curves at 1, 3 and 5 years postoperatively (**Figure 3J**). Subsequently, a risk score model was built for the evaluation of the prognostic predictive ability of SARDH expression in HCC. Results demonstrated that higher risk scores were associated with worse prognosis (**Figure 3K**), which was consistent with the results of K-M analysis (**Figure 3L**). The prognostic efficacy of the system was confirmed by time-dependent ROC curves at 1 year (AUC=0.662), 3 years (AUC=0.613), as well as 5 years (AUC=0.613) (**Figure 3M**).

### Identification of DEGs

**Figure 4:**
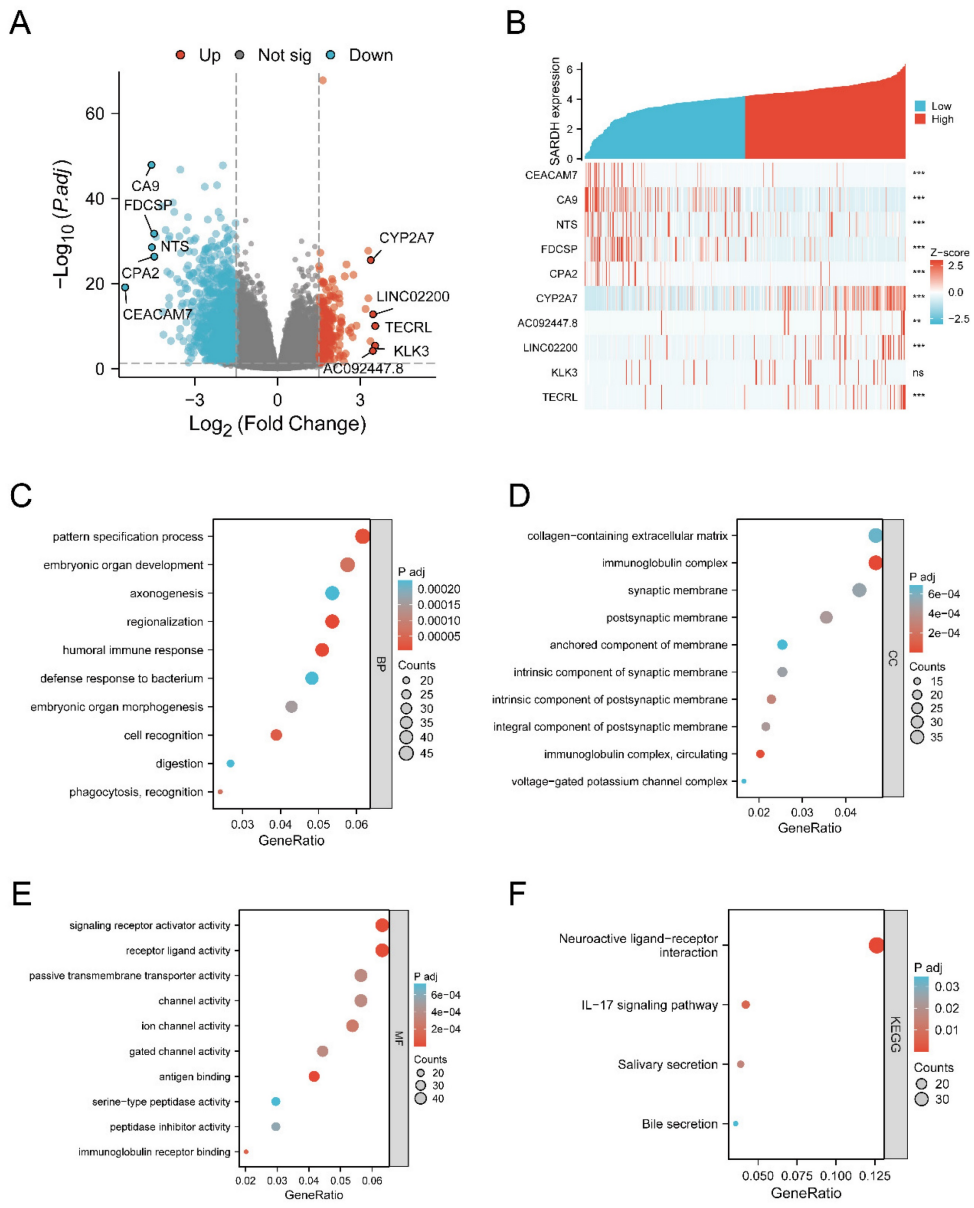
Analysis of differentially expressed genes and functional enrichment of SARDH in HCC. (A) Volcano plot of DEGs, with up-regulated genes in red and down-regulated genes in blue, and the top 5 up-regulated and down-regulated genes are labeled by name. (B) Heat map showing the correlation of the top up-regulated and down-regulated genes with SARDH expression. (C) Results of GO-BP enrichment analysis. (D) Results of GO-CC enrichment analysis. (E) Results of GO-MF enrichment analysis. (F) Results of KEGG enrichment analysis.

**Figure 5:**
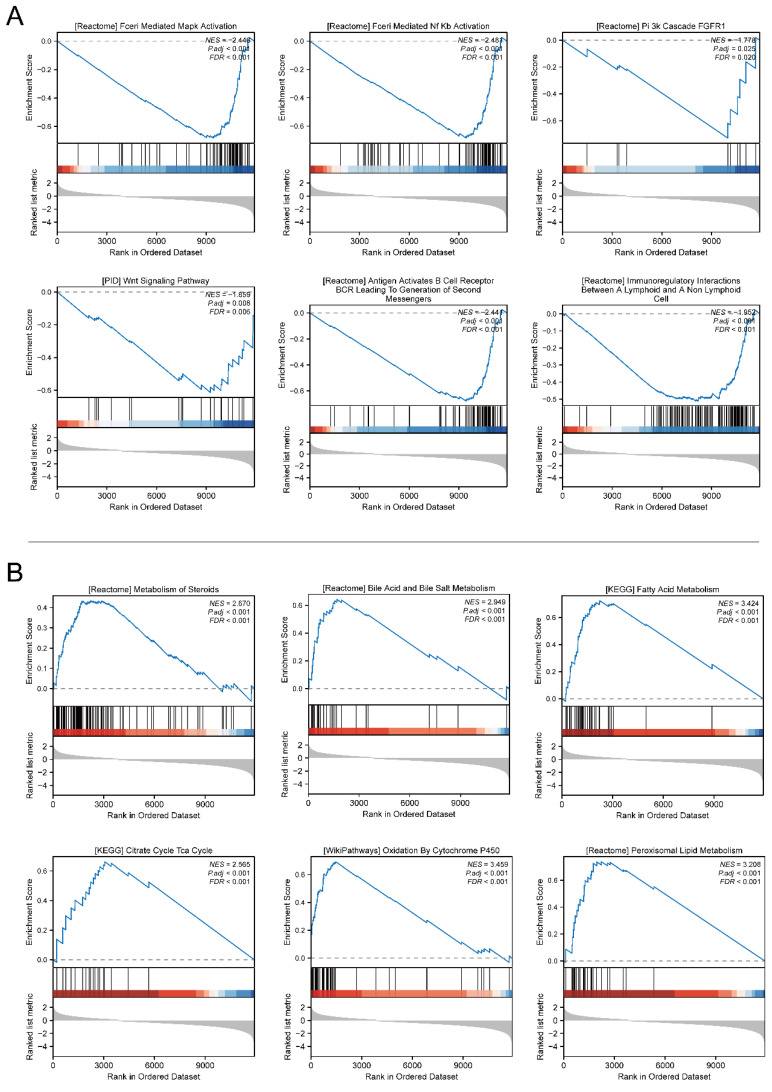
GSEA gene set enrichment results. (A) Enrichment results of GSEA gene set in SARDH low expression group. (B) Enrichment results of GSEA gene set in the SARDH high expression group.

We identified 1149 genes that were down-regulated and 232 genes that were up-regulated (**Figure 4A**). To further explore the relationship of SARDH expression with these DEGs, we analyzed the top 5 genes with significantly up- and down-regulated expression (**Figure 4B**). To explore the biological processes associated with SARDH, GO analysis, including biological processes, cellular components as well as molecular functions, was carried out. Results showed a correlation between SARDH expression and immune response (**Figure 4C-E**). Furthermore, KEGG analysis found these DEGs to be enriched in the IL-17 signaling pathway and bile secretion (**Figure 4F**). We performed GSEA analysis to explore the relationship between SARDH expression and various biological processes. High SARDH expression was found to be associated with citrate cycle (TCA cycle), Cytochrome P450 oxidation, bile acid, and metabolism of steroids, fatty acid, bile salt, and peroxisomal lipid. In contrast, low SARDH expression was associated with MAPK activation, Nf Kb activation, PI3k cascade FGFR1, WNT Signaling Pathway, B-cell activation and immune regulation by lymphocytes (**Figure 5**).

### SARDH expression is significantly associated with DNA methylation

**Figure 6:**
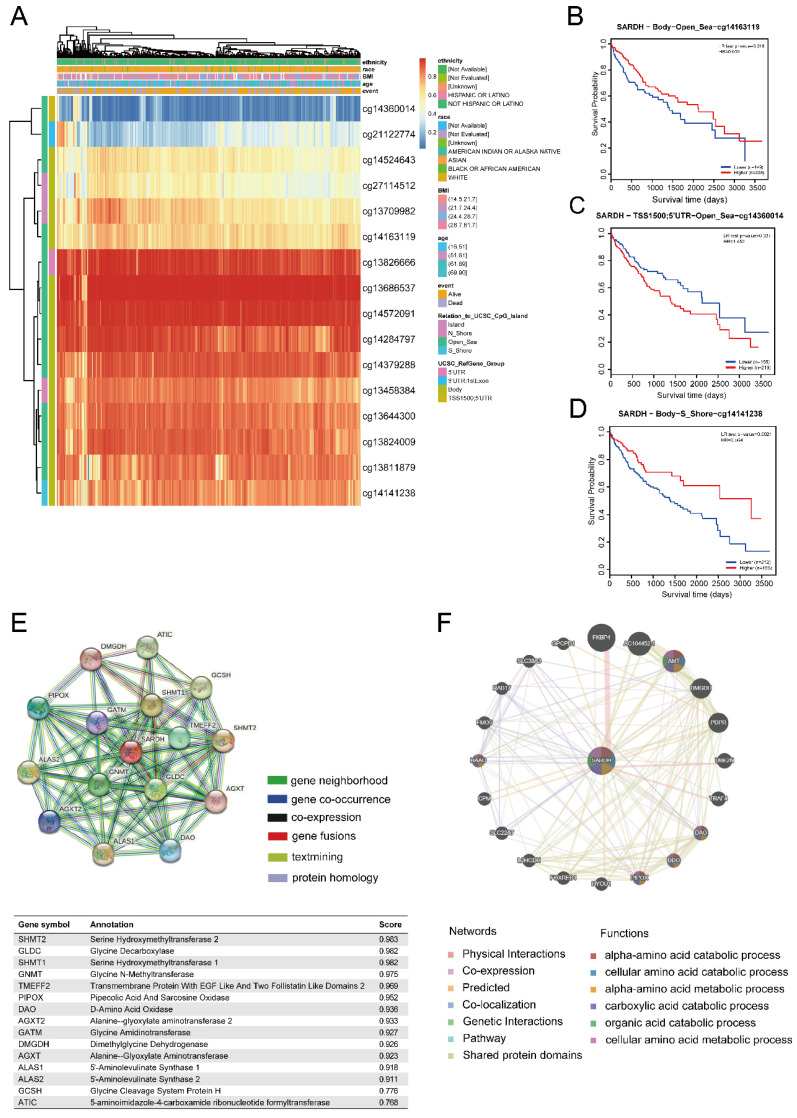
DNA methylation levels of SARDH and its impact on the prognosis of patients with HCC. (A) Correlation between SARDH expression levels and methylation levels and their prognosis. (B-D) Kaplan-Meier curves of 3 methylation sites in SARDH.STRING Protein Interaction Network and GeneMANIA Gene Interaction Network. (E) Protein- Protein interaction network (PPI) and annotation and correlation coefficients of 15 SARDH-related proteins. (F) Gene interaction network.

To examine the potential mechanisms of SARDH progression in HCC, we analyzed the relationship between SARDH expression and DNA methylation using the MethSurv database. Our findings showed that most of DNA methylation sites associated with SARDH in HCC were hypermethylated, and patients with SARDH hypermethylation had a lower OS compared to those with SARDH hypomethylation (**Figure 6A**). In addition, hypomethylation of methylation sites (cg14163119 and cg14141238) suggested worse prognosis, while hypermethylation of the methylation site (cg14360014) suggested worse prognosis (**Figure 6B-D**).

### Hub genes of SARDH

We explored the PPI network of SARDH with 15 other proteins using the STRING database (**Figure 6E**). The GeneMANIA database revealed that SARDH interacted with 20 potential target genes primarily related to the metabolism of amino acids (**Figure 6F**).

### SARDH may be involved in the regulation of Ferroptosis

**Figure 7:**
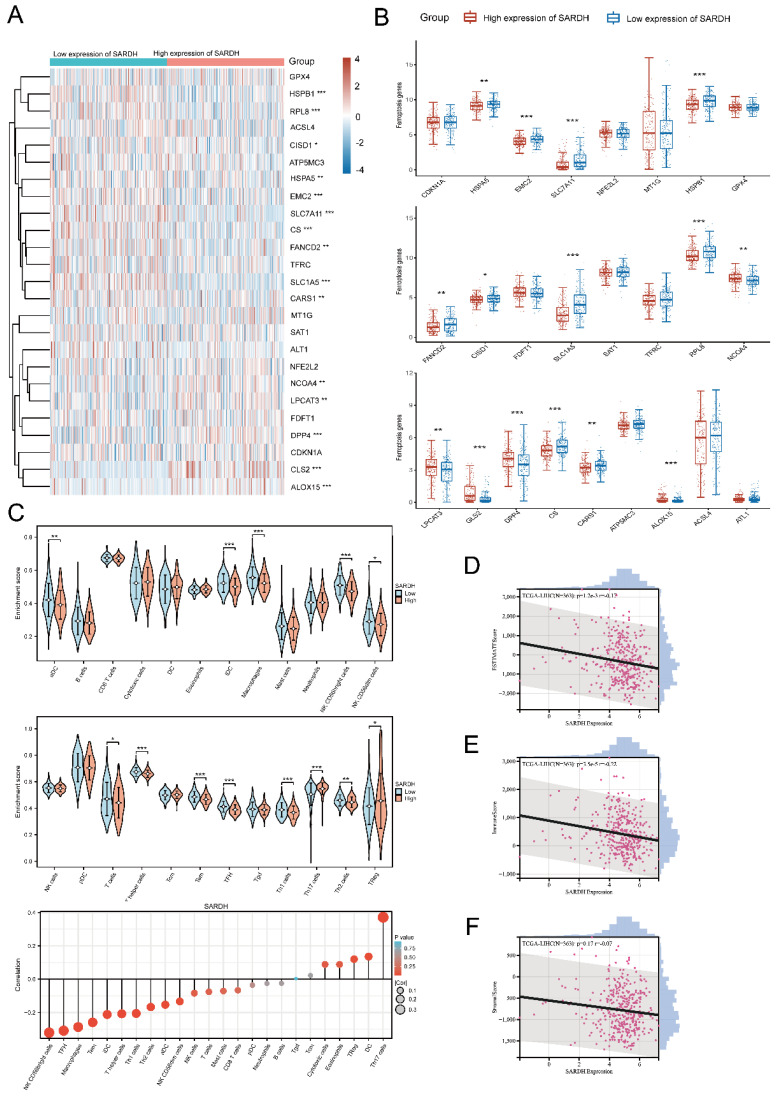
Correlation of SARDH with related genes in ferroptosis pathway. (A) Expression of ferroptosis-related genes in the high and low SARDH expression groups. (B) Heat map of the correlation between SARDH expression and ferroptosis-related genes. Correlation of SARDH with immune infiltration. (C) Comparison of immune infiltration levels of immune cells between high SARDH expression and low SARDH expression groups and correlation between SARDH expression and immune infiltration levels of 24 immune cells. (D)Correlation between SARDH expression and ESTIMATEScore. (E) Correlation between SARDH expression and ImmuneScore. (F) Correlation between SARDH expression and StromalScore. *p < 0.05; **p < 0.01; ***p < 0.001.

Ferroptosis has been found to exert a critical effect on the growth of HCC 32-34. To further explore the association between SARDH expression and 25 genes involving in ferroptosis pathway, we analyzed RNA-seq data from TCGA database 35. Spearman analysis revealed that SARDH expression was in a negative correlation with 10 genes and in a positive correlation with 5 genes in Ferroptosis pathway (**Figure 7A-B**).

### SARDH expression significantly correlated with immune infiltration

**Figure 8:**
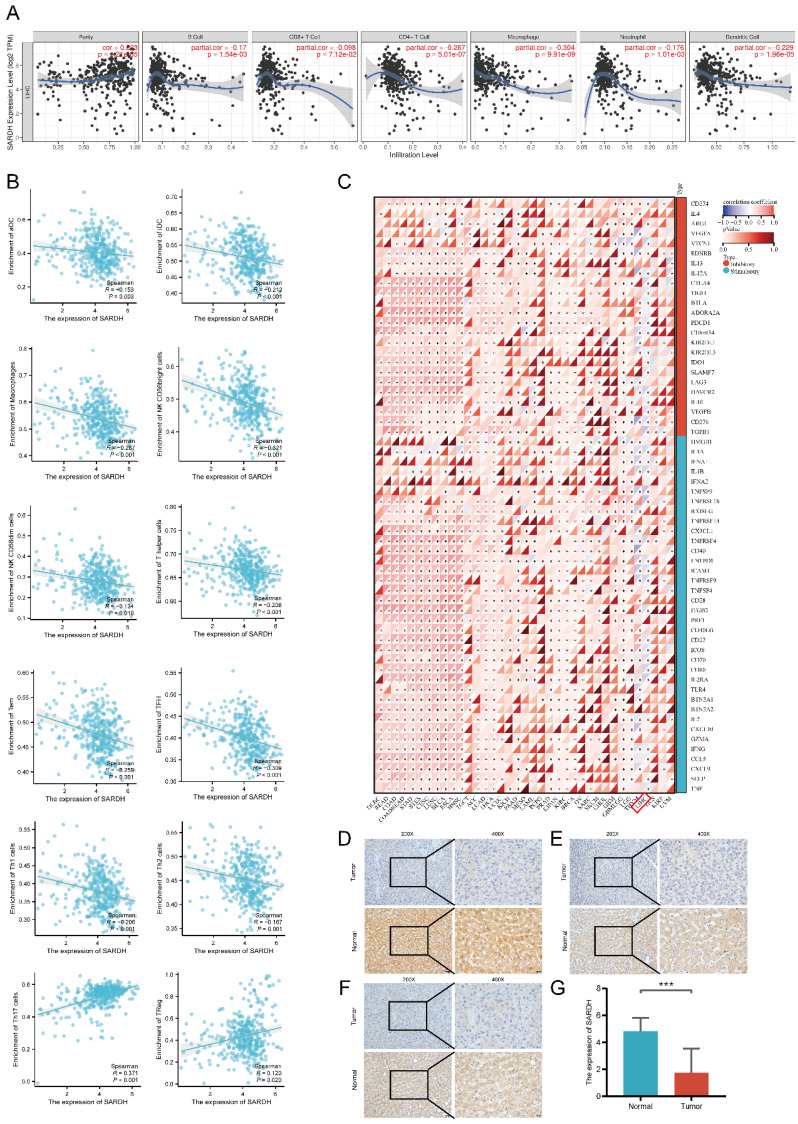
(A) Correlation of SARDH in TIMER with the infiltration status of 6 immune cells. (B) Scatter plot of the correlation between SARDH expression and immune infiltration level of immune cells. (C) Heat map of the correlation between SARDH expression and immune checkpoint expression. Typical immunohistochemical images of local hepatocellular carcinoma tissue and corresponding paraneoplastic tissue. (D-F) Typical immunohistochemical images. (G) Histogram of IHC results. ***p < 0.001.

To investigate the correlation of SARDH expression with immune infiltration, we determined the enrichment scores of 11 immune cells in groups with low / high SARDH expression. Our findings showed that compared to the high SARDH expression group, higher enrichment scores of 11 immune cells were observed in the low SARDH expression group. In contrast, compared to the high SARDH expression group, lower enrichment scores of 2 immune cells were observed in the low SARDH expression group. Furthermore, we observed that SARDH expression was in a negative correlation with the 11 immune cell infiltration levels, but in a positive correlation with the 2 immune cell infiltration levels (**Figure 7C, Figure 8B**). Additionally, we examined the association of SARDH expression with stromal scores, immune score, and estimation score, and results revealed a negative correlation of SARDH expression with immune score / estimate score (**Figure 7D-F**). The TIMER analysis revealed that SARDH expression was positively related with tumor purity (correlation =0.233) and negatively correlated with CD8+ T cells (r=-0.098), neutrophils (r =-0.176), Neutrophil (r =-0.176), CD4+ T cells (r =-0.267), dendritic cells (r=-0.229), B cells (r =-0.170), as well as macrophages (r =-0.304) (**Figure 8A**). Since immune checkpoint inhibitors (ICIs) are known to enhance the efficacy of immunotherapy for HCC, we studied the correlation between SARDH and 60 checkpoint genes using the TCGA database 36. Our findings showed that SARDH expression was negatively linked with 39 immune checkpoint gene expressions, including PD-1, CTLA4, as well as PD-L1, which were major suppressive immune checkpoints (**Figure 8C**). Anti-PD-1, anti-PD-L1 interaction and anti-CTLA4 treatment restored the functions of CD8+ and CD4+ T cells, thereby enhancing the efficacy of immunotherapy in patients with HCC 37.

### SARDH was downregulated in HCC

To verify the above findings, we collected 32 HCC tissues and corresponding paraneoplastic tissues. Our IHC results showed that SARDH was significantly downregulated in HCC (**Figure 8D-G**), which was consistent with the above analysis.

## Discussion

SARDH exerts a critical impact on the metabolism of sarcosine to glycine and coordinates with TMEFF2 in this process 19; while GNMT is a catalyzing enzyme that converts glycine to sarcosine 38. Together, they regulate the balance between sarcosine and glycine. The sarcosine has showed an oncogenic potential in prostate cancer and promotes cancer cell invasion. Knockdown of SARDH induces an invasive phenotype in benign prostate epithelial cells 39-41. In examination of urine samples collected from patients with HCC, GNMT was identified as a biomarker associated with HCC prognosis, and there was a strong correlation between sarcosine and GNMT 42. SARDH can lower sarcosine levels and acts as a tumor suppressor by binding with TMEFF2 in the 1-C metabolism pathway 19, 43. The sarcosine also influences the metabolism as well as metastatic ability of cancer cells in the TME 44. While glycine N-methyltransferases have been widely studied for their role in glycine metabolism, SARDH has not been fully investigated, particularly with respect to its molecular mechanisms and prognostic significance in HCC.

The pan-cancer analysis revealed that SARDH was significantly downregulated in 27 malignant tumors compared to their corresponding normal tissues, suggesting that SARDH may function as an oncogene. In HCC, SARDH mRNA expression was significantly down-regulated in both paired and unpaired tissue samples. ROC analysis of multiple databases showed that SARDH can potentially serve as a diagnostic biomarker with an AUC greater than 0.8. Additional analyses revealed that SARDH expression was significantly downregulated in HCC patients with advanced stage, mild / severe adjacent hepatic tissues, and G3/G4 histologic grade. SARDH was also demonstrated to be downregulated in HCC among HPA and CPTAC databases from the UALCAN website. These findings were consistent with the results of IHC analysis on local HCC samples. Based on these findings, it could be inferred that SARDH is an oncogenic factor in HCC. The low expression of SARDH suggested a worse prognosis in HCC, as well as in other cancers like prostate cancer, colorectal cancer and renal cell carcinoma 17-19. The study also constructed a nomogram that demonstrated SARDH had some predictive power for survival at 1, 3, and 5 years. These findings demonstrate that SARDH can work as a valid prognostic biomarker. GSEA was conducted and revealed that the low SARDH expression in HCC was linked to various critical signaling pathways, including Fceri Mediated MAPK activation, WNT Signaling pathway, Fceri mediated NF Kb activation, as well as PI3k- FGFR1 cascade. As evidenced in previous studies, the MAPK pathway is implicated in HCC progression and activated in over 50% of HCC patients, suggesting a poorer prognosis 45; the NF-κB pathway is predominantly linked to inflammation, cell death, hepatocellular injury, cirrhosis and hepatocellular carcinogenesis 46; the PI3K pathway represents an important signaling mechanism responsible for regulating metabolism, proliferation and apoptosis in HCC. PI3K pathway activation is significant in HCC progression and pivotal for its treatment 47; The WNT/β-linked protein signaling pathway in HCC is associated with multiple signaling cascades to regulate embryonic development, cell proliferation and differentiation, further driving the formation of HCC^48^, ^49^. These pathways have been shown to exert significant influence on the occurrence and progression of HCC, suggesting that SARDH might be potential as a novel biomarker for HCC.

DNA methylation is a way of regulating gene expression, primarily by suppressing gene expression 50. Our study revealed a significant relationship between low SARDH expression and its DNA hypermethylation, with SARDH hypermethylation indicating a poorer prognosis. While SARDH methylation has not yet been explored in the context of in HCC, it has been studied in renal cell carcinoma. SARDH hypermethylation was obviously related with the clinical aggressive characteristics and has been recognized as an important factor affecting recurrence-free survival in renal cell carcinoma 18.

TME is consisted by non-cellular components and immune cells, which are crucial hepatocarcinogenesis, metastasis and invasion of HCC cells 51. The role of TME on HCC has been reported in numerous studies. However, the effects of SARDH on HCC in the context of TME has not been fully investigated. SARDH expression was in a negative correaltion with infiltration levels of aDC, iDC, T cells, Tem, macrophages, NK CD56bright cells, T helper cells, NK CD56dim cells, TFH, Th1 cells, as well as Th2 cells in our study, suggesting that SARDH regulates immune cell infiltration to alter the TME and ultimately promote HCC progression. The combination of immunotherapy and targeted therapy may improve the prognosis in HCC patients 52. Immune checkpoints and immune infiltration complement each other in regulating the TME 53. In our study, SARDH expression was negatively associated with 39 immune checkpoint gene expressions, such as CD274, CTLA4, as well as PDCD1. The efficacy of ICIs in the treatment of advanced HCC has been demonstrated in previous studies 54. Taken together, our results suggest that SARDH may be potential for targeted therapies and immunotherapy, enhancing the efficacy of immunotherapy.

Recently, studies have found that ferroptosis-related genes in HCC were significantly related to immune regulation in HCC and can enhance the efficacy of ICIs 55, 56. Immune cell-mediated ferroptosis in the TME enhanced the efficacy of ICIs. Our study demonstrated that SARDH expression was negatively associated with HSPA5, EMC2, SLC7A11, HSPB1, FANCD2, CISD1, SLC1A5, RPL8, CS, CARS1, as well as SARDH but positively associated with NCOA4, LPCAT3, GLS2, DPP4, and ALOX15 in the ferroptosis pathway. These results suggest that studying the correlation between ferroptosis and immune infiltration could be a promising area for further investigation in HCC research.

There were still some shortcomings in our study. Our study focused on the analysis of online databases and IHC to discern THE prognostic value and pathogenesis of SARDH in HCC, including immune infiltration. However, our study lacked investigation into either the impact of SARDH on the biological function of HCC cells under in vitro conditions, or further exploration of potential molecular mechanisms of SARDH in HCC in vivo conditions.

In summary, SARDH can serve as a valuable biomarker for prognostic prediction and immunotherapy of HCC.

## Figures and Tables

**Table 1:** Relationship between SARDH expression and clinicopathological features in the TCGA database.

Characteristics	Low expression of SARDH	High expression of SARDH	p-value	X^2^
n	187	187		
Gender, n (%)			0.151	2.064
Female	67 (17.9%)	54 (14.4%)		
Male	120 (32.1%)	133 (35.6%)		
Missing	0	0		
Age, n (%)			0.878	0.023
<= 60	89 (23.9%)	88 (23.6%)		
> 60	97 (26%)	99 (26.5%)		
Missing	1	0		
Pathologic T stage, n (%)			0.002	14.611
T1	74 (19.9%)	109 (29.4%)		
T2	58 (15.6%)	37 (10%)		
T3	48 (12.9%)	32 (8.6%)		
T4	6 (1.6%)	7 (1.9%)		
Missing	1	2		
Pathologic N stage, n (%)			0.636	0.223
N0	128 (49.6%)	126 (48.8%)		
N1	3 (1.2%)	1 (0.4%)		
Missing	56	60		
Pathologic M stage, n (%)			0.645	0.211
M0	136 (50%)	132 (48.5%)		
M1	3 (1.1%)	1 (0.4%)		
Missing	48	54		
Pathologic stage, n (%)			0.002	14.849
Stage I	69 (19.7%)	104 (29.7%)		
Stage II	52 (14.9%)	35 (10%)		
Stage III	50 (14.3%)	35 (10%)		
Stage IV	4 (1.1%)	1 (0.3%)		
Missing	12	12		
AFP (ng/ml), n (%)			0.186	1.743
<= 400	99 (35.4%)	116 (41.4%)		
> 400	36 (12.9%)	29 (10.4%)		
Missing	52	42		
Adjacent hepatic tissue inflammation, n (%)			0.008	7.109
None	47 (19.8%)	71 (30%)		
Mild &Severe	68 (28.7%)	51 (21.5%)		
Missing	72	65		
Histologic grade, n (%)			0.022	9.597
G1	18 (4.9%)	37 (10%)		
G2	91 (24.7%)	87 (23.6%)		
G3	71 (19.2%)	53 (14.4%)		
G4	5 (1.4%)	7 (1.9%)		
Missing	2	3		

Note: AFP, alpha-fetoprotein.

**Table 2:** Relationship between SARDH expression and clinicopathological features in the GSE14520 database.

Characteristics	High expression of SARDH	Low expression of SARDH	p-value	X^2^
n	106	106		
Gender, n (%)			0.543	0.396
Female	16 (7.5%)	13 (6.1%)		
Male	90 (42.5%)	93 (43.9%)		
Missing	0	0		
Age(years), n (%)			0.725	0.123
<60	85 (40.1%)	87 (41%)		
≥60	21 (9.9%)	19 (9%)		
Missing	0	0		
Main Tumor Size (cm), n (%)			0.531	0.394
>5	39 (18.5%)	35 (16.6%)		
≤5	66 (31.3%)	71 (33.6%)		
Missing	1	0		
Multinodular, n (%)			0.614	0.254
No	85 (40.1%)	82 (38.7%)		
Yes	21 (9.9%)	24 (11.3%)		
Missing	0	0		
AFP (ng/ml), n (%)			0.355	0.855
>300	43 (20.6%)	51 (24.4%)		
≤300	60 (28.7%)	55 (26.3%)		
Missing	3	0		
Cirrhosis, n (%)			0.206	1.599
Yes	95 (44.8%)	100 (47.2%)		
No	11 (5.2%)	6 (2.8%)		
Missing	0	0		
TNM staging, n (%)			0.002	12.818
I	57 (26.9%)	32 (15.1%)		
II	28 (13.2%)	48 (22.6%)		
III	21 (9.9%)	26 (12.3%)		
Missing	0	0		

Note: AFP, alpha-fetoprotein.

## References

[1] Sung H, Ferlay J, Siegel RL, Laversanne M, Soerjomataram I, Jemal A (2021). Global Cancer Statistics 2020: GLOBOCAN Estimates of Incidence and Mortality Worldwide for 36 Cancers in 185 Countries. CA: a cancer journal for clinicians.

[2] Bray F, Ferlay J, Soerjomataram I, Siegel RL, Torre LA, Jemal A (2018). Global cancer statistics 2018: GLOBOCAN estimates of incidence and mortality worldwide for 36 cancers in 185 countries. CA: a cancer journal for clinicians.

[3] Chen S, Cao Z, Prettner K, Kuhn M, Yang J, Jiao L (2023). Estimates and Projections of the Global Economic Cost of 29 Cancers in 204 Countries and Territories From 2020 to 2050. JAMA Oncol.

[4] Li X, Ramadori P, Pfister D, Seehawer M, Zender L, Heikenwalder M (2021). The immunological and metabolic landscape in primary and metastatic liver cancer. Nat Rev Cancer.

[5] Anwanwan D, Singh SK, Singh S, Saikam V, Singh R (2020). Challenges in liver cancer and possible treatment approaches. Biochim Biophys Acta Rev Cancer.

[6] Zhou J, Sun H, Wang Z, Cong W, Wang J, Zeng M (2020). Guidelines for the Diagnosis and Treatment of Hepatocellular Carcinoma (2019 Edition). Liver Cancer.

[7] Wen T, Jin C, Facciorusso A, Donadon M, Han H-S, Mao Y (2018). Multidisciplinary management of recurrent and metastatic hepatocellular carcinoma after resection: an international expert consensus. Hepatobiliary Surg Nutr.

[8] Keenan BP, Fong L, Kelley RK (2019). Immunotherapy in hepatocellular carcinoma: the complex interface between inflammation, fibrosis, and the immune response. J Immunother Cancer.

[9] Ren Z, Ducreux M, Abou-Alfa GK, Merle P, Fang W, Edeline J (2023). Tislelizumab in Patients with Previously Treated Advanced Hepatocellular Carcinoma (RATIONALE-208): A Multicenter, Non-Randomized, Open-Label, Phase 2 Trial. Liver Cancer.

[10] Qin S, Ren Z, Feng Y-H, Yau T, Wang B, Zhao H (2021). Atezolizumab plus Bevacizumab versus Sorafenib in the Chinese Subpopulation with Unresectable Hepatocellular Carcinoma: Phase 3 Randomized, Open-Label IMbrave150 Study. Liver Cancer.

[11] Kelley RK, Sangro B, Harris W, Ikeda M, Okusaka T, Kang Y-K (2021). Safety, Efficacy, and Pharmacodynamics of Tremelimumab Plus Durvalumab for Patients With Unresectable Hepatocellular Carcinoma: Randomized Expansion of a Phase I/II Study. J Clin Oncol.

[12] Brar G, Greten TF, Brown ZJ (2018). Current frontline approaches in the management of hepatocellular carcinoma: the evolving role of immunotherapy. Therap Adv Gastroenterol.

[13] Iñarrairaegui M, Melero I, Sangro B (2018). Immunotherapy of Hepatocellular Carcinoma: Facts and Hopes. Clin Cancer Res.

[14] Wagner C, Briggs WT, Cook RJ (1985). Inhibition of glycine N-methyltransferase activity by folate derivatives: implications for regulation of methyl group metabolism. Biochem Biophys Res Commun.

[15] Bergeron F, Otto A, Blache P, Day R, Denoroy L, Brandsch R (1998). Molecular cloning and tissue distribution of rat sarcosine dehydrogenase. Eur J Biochem.

[16] Yeo EJ, Wagner C (1994). Tissue distribution of glycine N-methyltransferase, a major folate-binding protein of liver. Proc Natl Acad Sci U S A.

[17] He H, Chen E, Lei L, Yan B, Zhao X, Zhu Z (2019). Alteration of the tumor suppressor SARDH in sporadic colorectal cancer: A functional and transcriptome profiling-based study. Mol Carcinog.

[18] Mazdak M, Tezval H, Callauch JC, Dubrowinskaja N, Peters I, Bokemeyer C (2019). DNA methylation of sarcosine dehydrogenase (SARDH) loci as a prognosticator for renal cell carcinoma. Oncol Rep.

[19] Green T, Chen X, Ryan S, Asch AS, Ruiz-Echevarría MJ (2013). TMEFF2 and SARDH cooperate to modulate one-carbon metabolism and invasion of prostate cancer cells. Prostate.

[20] Lim SO, Park S-J, Kim W, Park SG, Kim H-J, Kim Y-I (2002). Proteome analysis of hepatocellular carcinoma. Biochem Biophys Res Commun.

[21] Uhlén M, Fagerberg L, Hallström BM, Lindskog C, Oksvold P, Mardinoglu A (2015). Proteomics. Tissue-based map of the human proteome. Science.

[22] Chandrashekar DS, Karthikeyan SK, Korla PK, Patel H, Shovon AR, Athar M (2022). UALCAN: An update to the integrated cancer data analysis platform. Neoplasia.

[23] Menyhárt O, Nagy Á, Győrffy B (2018). Determining consistent prognostic biomarkers of overall survival and vascular invasion in hepatocellular carcinoma. R Soc Open Sci.

[24] Tang Z, Kang B, Li C, Chen T, Zhang Z (2019). GEPIA2: an enhanced web server for large-scale expression profiling and interactive analysis. Nucleic Acids Res.

[25] Modhukur V, Iljasenko T, Metsalu T, Lokk K, Laisk-Podar T, Vilo J (2018). MethSurv: a web tool to perform multivariable survival analysis using DNA methylation data. Epigenomics.

[26] Szklarczyk D, Gable AL, Nastou KC, Lyon D, Kirsch R, Pyysalo S (2021). The STRING database in 2021: customizable protein-protein networks, and functional characterization of user-uploaded gene/measurement sets. Nucleic Acids Res.

[27] Franz M, Rodriguez H, Lopes C, Zuberi K, Montojo J, Bader GD (2018). GeneMANIA update 2018. Nucleic Acids Res.

[28] Yoshihara K, Shahmoradgoli M, Martínez E, Vegesna R, Kim H, Torres-Garcia W (2013). Inferring tumour purity and stromal and immune cell admixture from expression data. Nat Commun.

[29] Bindea G, Mlecnik B, Tosolini M, Kirilovsky A, Waldner M, Obenauf AC (2013). Spatiotemporal dynamics of intratumoral immune cells reveal the immune landscape in human cancer. Immunity.

[30] Obuchowski NA, Bullen JA (2018). Receiver operating characteristic (ROC) curves: review of methods with applications in diagnostic medicine. Phys Med Biol.

[31] Zhou X, Huang J-M, Li T-M, Liu J-Q, Wei Z-L, Lan C-L (2022). Clinical Significance and Potential Mechanisms of ATP Binding Cassette Subfamily C Genes in Hepatocellular Carcinoma. Front Genet.

[32] Iseda N, Itoh S, Toshida K, Tomiyama T, Morinaga A, Shimokawa M (2022). Ferroptosis is induced by lenvatinib through fibroblast growth factor receptor-4 inhibition in hepatocellular carcinoma. Cancer Sci.

[33] Zhang L, Li X-M, Shi X-H, Ye K, Fu X-L, Wang X (2023). Sorafenib triggers ferroptosis via inhibition of HBXIP/SCD axis in hepatocellular carcinoma. Acta Pharmacol Sin.

[34] Chen S, Xia H, Sheng L (2023). WTAP-mediated m6A modification on circCMTM3 inhibits hepatocellular carcinoma ferroptosis by recruiting IGF2BP1 to increase PARK7 stability. Dig Liver Dis.

[35] Liu Z, Zhao Q, Zuo Z-X, Yuan S-Q, Yu K, Zhang Q (2020). Systematic Analysis of the Aberrances and Functional Implications of Ferroptosis in Cancer. iScience.

[36] Thorsson V, Gibbs DL, Brown SD, Wolf D, Bortone DS, Ou Yang T-H (2018). The Immune Landscape of Cancer. Immunity.

[37] Llovet JM, Castet F, Heikenwalder M, Maini MK, Mazzaferro V, Pinato DJ (2022). Immunotherapies for hepatocellular carcinoma. Nat Rev Clin Oncol.

[38] Luka Z, Mudd SH, Wagner C (2009). Glycine N-methyltransferase and regulation of S-adenosylmethionine levels. J Biol Chem.

[39] Khan AP, Rajendiran TM, Ateeq B, Asangani IA, Athanikar JN, Yocum AK (2013). The role of sarcosine metabolism in prostate cancer progression. Neoplasia.

[40] Sreekumar A, Poisson LM, Rajendiran TM, Khan AP, Cao Q, Yu J (2009). Metabolomic profiles delineate potential role for sarcosine in prostate cancer progression. Nature.

[41] Zabala-Letona A, Arruabarrena-Aristorena A, Fernandez-Ruiz S, Viera C, Carlevaris O, Ercilla A (2022). PI3K-regulated Glycine N-methyltransferase is required for the development of prostate cancer. Oncogenesis.

[42] Oh S, Jo S, Kim HS, Mai V-H, Endaya B, Neuzil J (2023). Chemical Biopsy for GNMT as Noninvasive and Tumorigenesis-Relevant Diagnosis of Liver Cancer. Anal Chem.

[43] Chen X, Overcash R, Green T, Hoffman D, Asch AS, Ruiz-Echevarría MJ (2011). The tumor suppressor activity of the transmembrane protein with epidermal growth factor and two follistatin motifs 2 (TMEFF2) correlates with its ability to modulate sarcosine levels. J Biol Chem.

[44] Stepka P, Vsiansky V, Raudenska M, Gumulec J, Adam V, Masarik M (2021). Metabolic and Amino Acid Alterations of the Tumor Microenvironment. Curr Med Chem.

[45] Delire B, Stärkel P (2015). The Ras/MAPK pathway and hepatocarcinoma: pathogenesis and therapeutic implications. Eur J Clin Invest.

[46] Luedde T, Schwabe RF (2011). NF-κB in the liver-linking injury, fibrosis and hepatocellular carcinoma. Nat Rev Gastroenterol Hepatol.

[47] Sun EJ, Wankell M, Palamuthusingam P, McFarlane C, Hebbard L (2021). Targeting the PI3K/Akt/mTOR Pathway in Hepatocellular Carcinoma. Biomedicines.

[48] He S, Tang S (2020). WNT/β-catenin signaling in the development of liver cancers. Biomed Pharmacother.

[49] Xu C, Xu Z, Zhang Y, Evert M, Calvisi DF, Chen X (2022). β-Catenin signaling in hepatocellular carcinoma. J Clin Invest.

[50] Moore LD, Le T, Fan G (2013). DNA methylation and its basic function. Neuropsychopharmacology.

[51] Yang JD, Nakamura I, Roberts LR (2011). The tumor microenvironment in hepatocellular carcinoma: current status and therapeutic targets. Semin Cancer Biol.

[52] Cheng A-L, Qin S, Ikeda M, Galle PR, Ducreux M, Kim T-Y (2022). Updated efficacy and safety data from IMbrave150: Atezolizumab plus bevacizumab vs. sorafenib for unresectable hepatocellular carcinoma. J Hepatol.

[53] Giraud J, Chalopin D, Blanc J-F, Saleh M (2021). Hepatocellular Carcinoma Immune Landscape and the Potential of Immunotherapies. Front Immunol.

[54] Zongyi Y, Xiaowu L (2020). Immunotherapy for hepatocellular carcinoma. Cancer Lett.

[55] Xu Z, Peng B, Liang Q, Chen X, Cai Y, Zeng S (2021). Construction of a Ferroptosis-Related Nine-lncRNA Signature for Predicting Prognosis and Immune Response in Hepatocellular Carcinoma. Front Immunol.

[56] Wei R, Qiu H, Xu J, Mo J, Liu Y, Gui Y (2020). Expression and prognostic potential of GPX1 in human cancers based on data mining. Ann Transl Med.

